# Pure Zirconium: Type II Nodal Line and Nodal Surface States

**DOI:** 10.3389/fchem.2020.585753

**Published:** 2020-09-23

**Authors:** Li Zhang, Kai Wang

**Affiliations:** ^1^Changchun Institute of Technology, Changchun, China; ^2^Engineering Research Center (ERC), Harbin Medical University, Harbin, China; ^3^Nanoscience and Technology Center, The Fourth Medical College of Harbin Medical University, Harbin, China

**Keywords:** pure Zr, nodal line state, nodal surface state, electronics structures, first-principle prediction

## Abstract

Type II nodal line states have novel properties, such as direction-reliant chiral anomalies and high anisotropic negative magneto-resistance. These type II nodal line states have been widely investigated. Compared to nodal line materials, there are far fewer proposed nodal surface materials, and furthermore, a very recent challenge is to find a realistic material that co-exhibits both nodal line and nodal surface states. In this manuscript, we present the study of the electronic and topological states of pure zirconium within the density functional theory. We found that pure Zr is an interesting material that rarely exhibits both the type II nodal line state (in k_z_ = 0 plane) and nodal surface state (in k_z_ = π plane). The nontrivial topological states are explained based on the orbital-resolved band structures. Our study shows that pure Zr can serve as a new platform to investigate the interplay between the nodal line state and the nodal surface state.

## Introduction

Topological semimetals and topological metals (Fang et al., 2016; Yan and Felser, 2017; Schoop et al., 2018; Zhou et al., 2018; Gao et al., 2019; Hu et al., 2019; Klemenz et al., 2019; Pham et al., 2019; Xie et al., 2019; Yi et al., 2019) have been widely investigated because they can be regarded as good candidates for use in the areas of spintronics and quantum computers. Weyl and Dirac materials (Ouyang et al., 2016; Zhong et al., 2016; Zhou et al., 2016; Liu et al., 2017; Fu et al., 2018; Meng et al., 2019, 2020a; Zhang et al., 2020), which host 2-fold and fourfold degenerate band-crossing points, have been explored in real materials and their exotic properties have been confirmed in experiments. Moving forward, a series of three-dimension materials, with 1D and 2D band crossing points, have been predicted to be nodal line semimetals/metals (Phillips and Aji, 2014; Gan et al., 2017; Jin et al., 2017, 2019, 2020; Lu et al., 2017; Yang et al., 2017; Chen et al., 2018; Gao et al., 2018; Liu et al., 2018) and nodal surface semimetals/metals (Wu et al., 2018; Zhang et al., 2018; Wang et al., 2020), respectively.

Moreover, topological semimetals/metals can also be classified by the tilting degree of the fermion cone. Hence, Weyl materials can be roughly divided into two main categories, namely, type I Weyl semimetals/metals (Osterhoudt et al., 2019) where the two bands have opposite velocities and type II Weyl materials (Soluyanov et al., 2015; Ma et al., 2019) where the two bands have the same velocity. Type II Weyl materials are expected to exhibit many interesting features (Koepernik et al., 2016; Yu et al., 2016; Sharma et al., 2017), such as signals in magneto-oscillations, anisotropic chiral anomalies, and an unusual magneto-response.

Besides the two types of Weyl materials, a third type exists, named hybrid Weyl materials (Alisultanov, 2018), in which one Weyl point is type I while the other one is type II. Similar to Weyl materials, nodal line materials are composed of numerous band crossing points and they can also be classified as type I, type II, and hybrid types on the basis of the band dispersion around the band crossing points.

Nodal surface materials have been proposed in some 3D materials with different families, such as the Ti_3_Al family (Zhang et al., 2018), BaVS_3_ family (Liang et al., 2016), and HfIr_3_B_4_ (Wang et al., 2020). The predicted nodal surface materials are far fewer, compared to nodal line and nodal point materials. What is more, up to now, there has been no experimental verification of nodal surface materials. Noted that the nodal surface properties can be predicted among magnetic materials due to the bands for each spin channel can be effectively seen as a spinless system with a chosen spin polarization axis. Therefore, nodal surface materials can be seen as good candidates for using in spintronic.

In this manuscript, we aim to present a first-principle study of the electronic structures and the topological signatures of a new metal (i.e., pure Zr) co-featuring the type II nodal line state and the nodal surface state. Importantly, P6_3_/mmc Zr is a realistic material and its experimental lattice constants are a = b = 3.232 Å; c = 5.147 Å, respectively (Wyckoff, 1963). Our results provide a realistic material platform for exploring the fundamental physics of type II nodal line and nodal surface states, and their hybridization.

## Computational Details

We performed this study by the following main steps: (i) We investigated the most stable configuration by volume optimization and calculation of lattice parameters for P6_3_/mmc Zr, (ii) We computed band structures, including the 2D band structure along Γ-M-K-Γ-A-L-H-A high-symmetry points and the orbital-resolved band structures. These two investigations were completed with the Vienna ab initio Simulation Package (Sun et al., 2003). Volume optimizations and lattice parameter estimations were carried out with the generalized gradient approximation (GGA) (Perdew et al., 1996) of the Perdew–Burke–Ernzerhof (PBE) functional (Perdew et al., 1998). The cutoff energy was set as 600 eV. The Brillouin zone was sampled by a Monkhorst–Pack *k*-mesh with a size of 11 × 11 × 6. The self-consistent field convergence for the total energy and the force variation were set as 1 × 10^−6^ eV and 0.00001 eV·Å^−1^, respectively.

**Figure 1:**
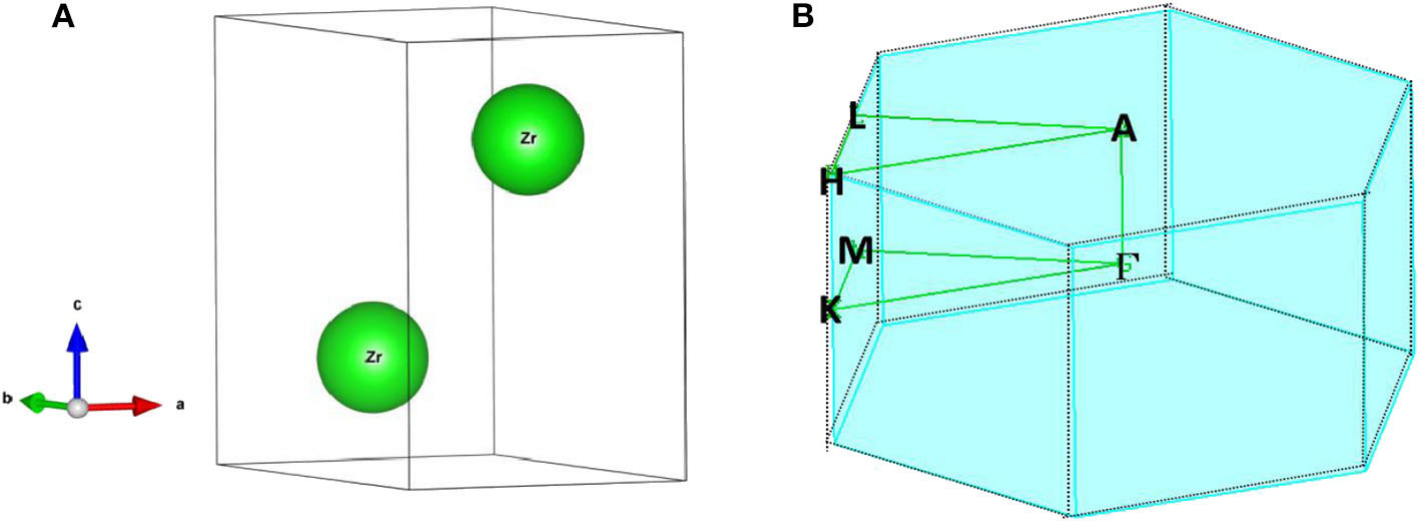
**(A)** Primitive cell and **(B)** the bulk Brillouin zone of pure Zr.

Experimentally, Zr is a realistic material and it naturally shares a hexagonal phase, with 194 space group numbers, a P6_3_/mmc space group, and a series of ICSDs (such as 653524, 653525, 653528, 653529)^1^. The crystal structure of Zr was fully relaxed before the band-structure calculation. The crystal model as well as the atomic positions were determined and are shown in Figure 1A. From this, one can see that this crystal model contains two Zr atoms; one located at the (0.6666, 0.3333, 0.75) position, and the other located at the (0.3333, 0.6666, 0.25) position. The achieved optimized lattice constants are a = b = 3.234 Å; c = 5.161 Å, respectively, which are in good agreement with the experimental ones (Wyckoff, 1963). The calculated electronic density of states of Zr is shown in Figure S1, the metallic properties of Zr can be observed due to a large peak appears around the Fermi level.

## Results and Discussion

**Figure 2:**
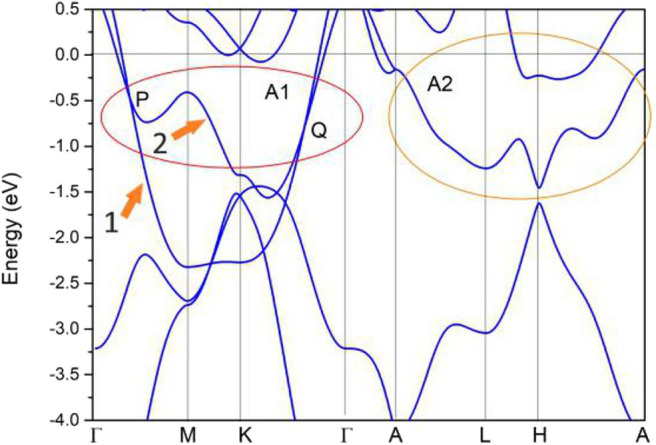
Band structure of pure Zr *via* the GGA-PBE method. A series of band crossing points can be found near the Fermi level, and these band crossing points are divided into two regions (i.e., region A1 and region A2).

We first consider the electronic band structure of pure Zr in the absence of spin-orbit coupling. The GGA-PBE result for the band structure along Γ-M-K-Γ-A-L-H-A high-symmetry points (see Figure 1B) is exhibited in Figure 2. From this figure, one can see the metallic behavior of this material with some obvious band crossing points. These band crossing points are formed by the overlapping between band 1 (labeled as 1) and band 2 (labeled as 2). These two bands belong to the irreducible representations *A*_1_ and *B*_2_ of the *C*_2*v*_ symmetry. Furthermore, these band crossing points are mainly located at two regions, named as A1 and A2, respectively. In Figure S2, the band structure of Zr metal under experimental lattice constants is also computed via GGA method and one can see that these band-crossing points are still retained.

In the A1 region, two band crossing points (i.e., P and Q) can be found along the Γ-M and M-K paths, respectively. All of these band crossing points are located quite close to the Fermi level. Because the pure Zr system enjoys spatial inversion and time reversal symmetries, the P and Q band crossing points belong to a nodal line instead of isolated points (Jin et al., 2019). Moreover, the Γ-M and M-K paths are situated in the mirror-invariant plane *k*_*z*_ = 0, which can protect a nodal line.

**Figure 3:**
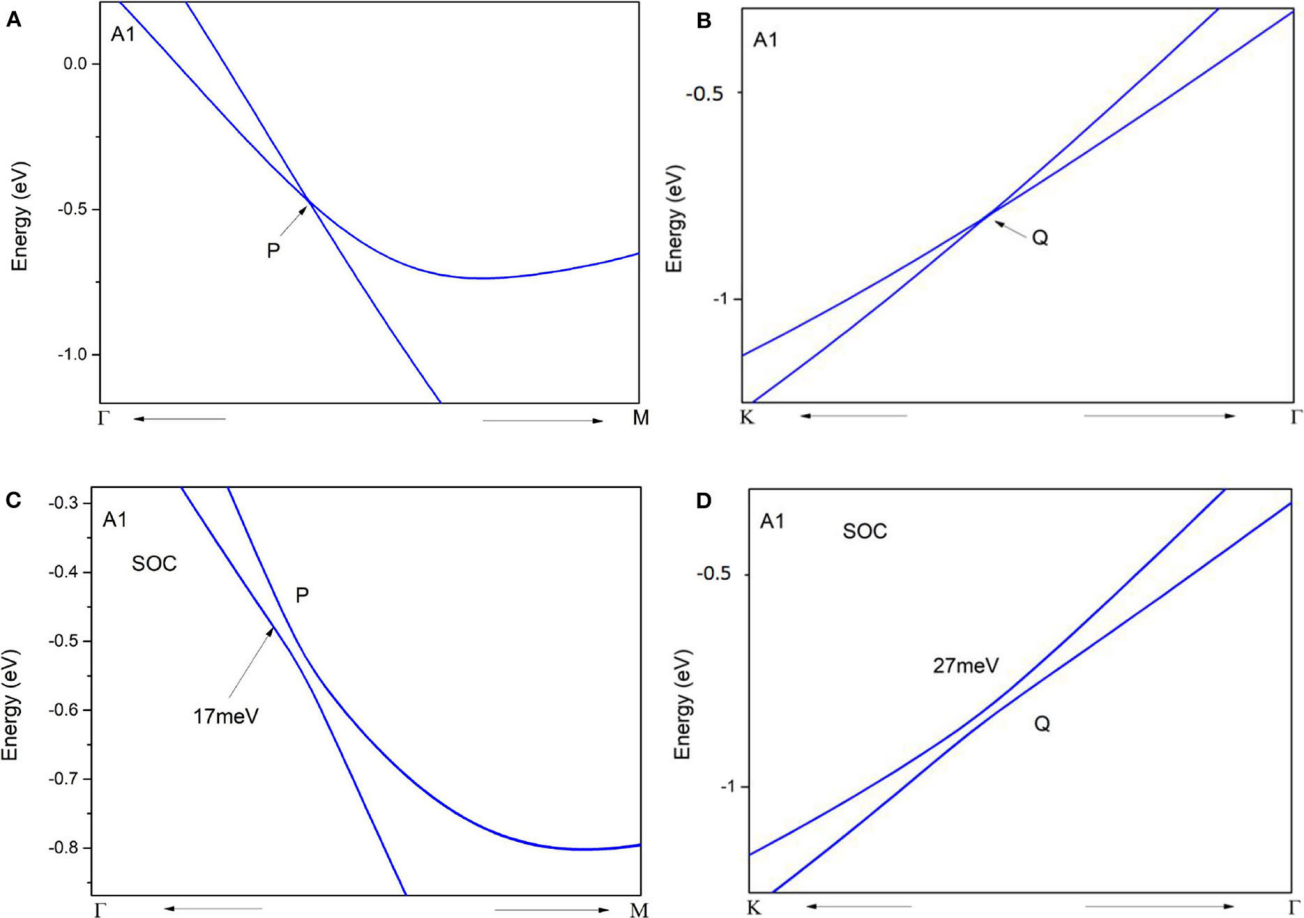
Enlarged band structures of pure Zr *via* the GGA-PBE method along Γ-M **(A,C)** and M-K **(B,D)** paths, respectively. The band structures calculated in **(A–D)** do not have spin–orbit coupling (have spin–orbit coupling).

**Figure 4:**
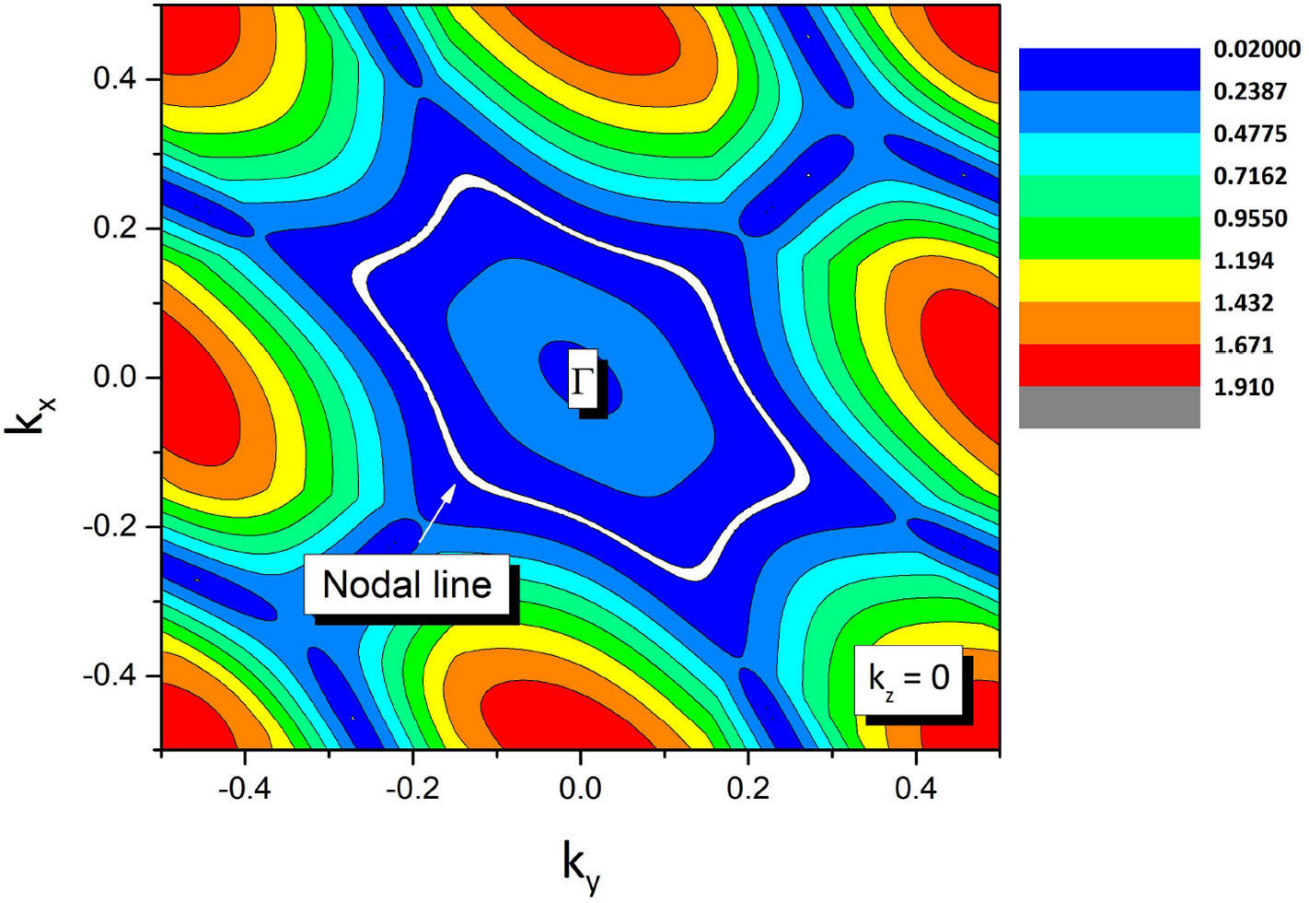
The shape of the nodal line at the k_z_ = 0 plane. The Γ-centered nodal line is marked as a white line.

The enlarged band structures (in the absence of spin-orbital coupling) around P and Q band crossing points are given in Figures 3A,B, respectively. Around these band crossing points, the band structures host a large linear energy region (see Figures 3A,B). Based on the slopes of the involved bands in crystal momentum space around the band crossing points P and Q, one can see that they belong to type II. To determine whether P and Q band crossing points are isolated nodal points or belong to a nodal line, the shape of the nodal line at the *k*_*z*_ = 0 plane is exhibited in Figure 4. From this figure, a Γ-centered nodal line (highlighted by the white line) can be obviously observed at the *k*_*z*_ = 0 plane.

One should note that the nodal line state is protected by two independent mechanisms for Zr system: one is the spatial inversion and the time reversal symmetries; the other one is mirror symmetry *M*_*z*_, because the two crossing bands have opposite mirror eigenvalues. Therefore, the nodal line state in the Zr system is robust because Zr hosts two kinds of symmetry-protected mechanisms. That is, if we break only one kind of symmetry protection, the nodal line state will be retained. The same symmetry protection mechanism has also been reported in previous work, such as TiB_2_ with the type I nodal line state (Zhang et al., 2017).

The SOC effect was also taken into consideration in the Zr system in region A1, and the calculated band structures around P and Q band crossing points are given in Figures 3C,D, respectively. From these figures, one can see that the SOC-induced gaps around P and Q are ~17 and ~27 meV, respectively. We noted that these opened gaps around the band crossing points are indeed small and these values of Zr are smaller than the values of some previously proposed nodal line materials (Meng et al., 2020b), such as Cu_3_NPd (60–100 meV), CaAgBi (80–140 meV), BaSn_2_ (60–160 meV).

**Figure 5:**
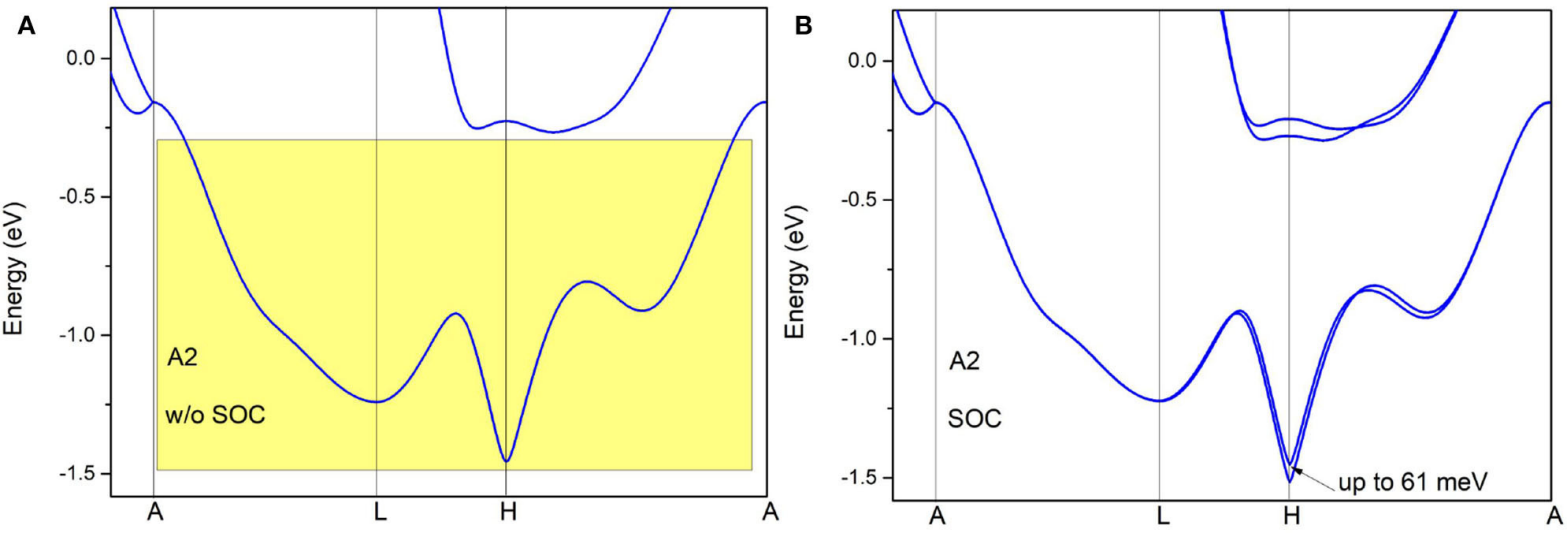
Band structures of pure Zr *via* the GGA-PBE method along A-L-H-A paths. The band structures calculated in **(A,B)** are without spin–orbit coupling (with spin–orbit coupling).

The band structure of pure Zr along A-L-H-A paths is given without SOC in Figure 5A. In region A2, there are two bands linearly crossing at the A point, and they then become degenerate in plane *k*_*z*_ = π forming a nodal surface. The nodal surface state is essential because it can be indicated by symmetry (i.e., the non-symmorphic *S*_2*z*_ and the time reversal symmetries *T*). Consider the twofold screw rotation *S*_2*z*_ in pure Zr, *S*_2*z*_ *(x,y,z)*→*(-x,-y,z*+*1/2)*. In the *k*_*z*_ = π plane, each k point is invariant under *S*_2*z*_*T*. Because *(S*_2*z*_*T)*^2^ = *T*_001_ = on plane *k*_*z*_ = π, the bands on this plane have a Kramer-like degeneracy. A nodal surface can be also understood as a result of Kramer degeneracy.

**Figure 6:**
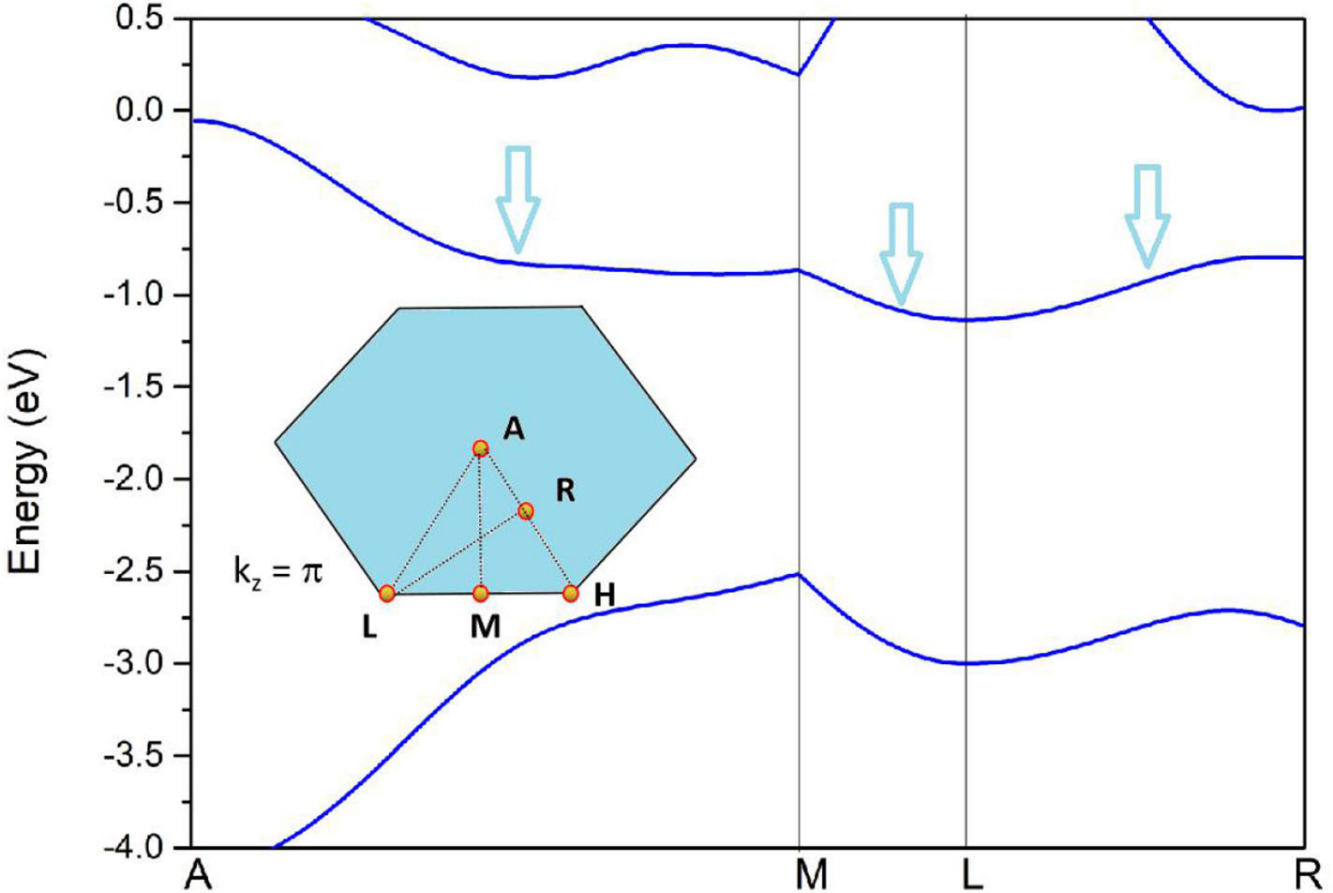
Band structures of pure Zr *via* the GGA-PBE method along A-M-L-R paths. The insert figure is selected k-paths at the k_z_ = π plane.

To further confirm the nodal surface state in the *k*_*z*_ = π plane, the band structure along the A-M-L-R paths (see the insert figure in Figure 6) is given in Figure 6. From it, one can see that the two bands are also degenerated with each other in this plane, which results in a nodal surface state in plane *k*_*z*_ = π. The effect of spin–orbit coupling (SOC) on the electronic structure is examined and the results of band structure along A-L-H-A (with SOC) are given in Figure 5B. The values of the SOC-induced gaps along A-L-H-A directions are up to 61 meV. The gaps in Zr are smaller than those in some topological materials (Meng et al., 2020b), such as Cu_3_NPd (60–100 meV), and BaSn_2_ (60–160 meV).

**Figure 7:**
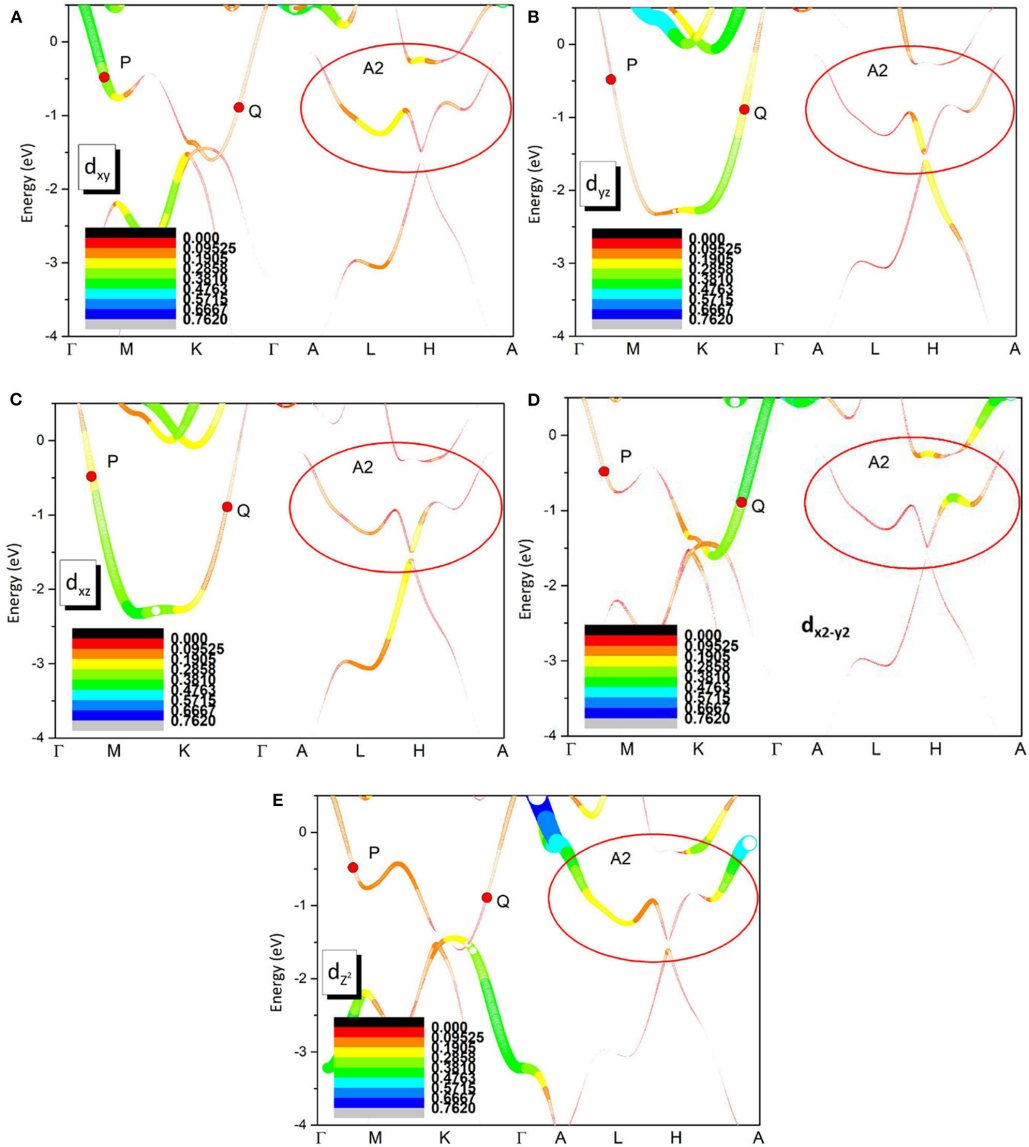
Orbital resolved band structures of pure Zr *via* the GGA-PBE method along A-M-L-R paths. **(A–E)** are for Zr-d_xy_, Zr-d_yz_, Zr-d_xz_, Zr-, Zr- orbitals, respectively.

Finally, the orbital-resolved band structures of Zr-d_xy_, Zr-d_yz_, Zr-d_xz_, ${\text{Zr-d}}_{{\text{x}}^{2}-{\text{y}}^{2}}$, ${\text{Zr-d}}_{{\text{z}}^{2}}$ orbitals are given in Figure 7. One can see that band 1 (see Figure 2) is mainly coming from the hybridization between the Zr-d_yz_ and Zr-d_xz_ orbitals. However, band 2 forms the hybridization among Zr-d_xy_, ${\text{Zr-d}}_{{\text{x}}^{2}-{\text{y}}^{2}}$, ${\text{Zr-d}}_{{\text{z}}^{2}}$ orbitals. In the A1 region, as shown in Figure 7, the band crossing point P is mainly coming from Zr-d_xz_ and Zr-d_xy_ orbitals, however, the band crossing point Q is mainly arising from the Zr-d_yz_ and ${\text{Zr-d}}_{{\text{x}}^{2}-{\text{y}}^{2}}$ orbitals. In region A2, the band crossing points of the surface states (*k*_*z*_ = π plane) are dominated by the ${\text{Zr-d}}_{{\text{z}}^{2}}$ orbital, however, the contribution of other orbitals of Zr atom cannot be ignored.

## Conclusions

In conclusion, based on symmetry analysis and first-principle calculations, we have shown a realistic material, pure Zr, which features a type II nodal line state at the *k*_*z*_ = 0 plane and a nodal surface state at the *k*_*z*_ = π plane when the SOC effect is ignored. The nodal line state at the *k*_*z*_ = 0 plane is protected by the spatial inversion, time reversal, and horizontal mirror symmetries. The nodal surface state at the *k*_*z*_ = π plane is protected by the screw rotation and time reversal symmetries. The effect of SOC on the topological nodal line and nodal surface states was tested and the SOC-induced band gaps for both A1 and A2 regions were found to be smaller than 61 meV.

The orbital-resolved band structures of Zr-d_xy_, Zr-d_yz_, Zr-d_xz_, ${\text{Zr-d}}_{{\text{x}}^{2}-{\text{y}}^{2}}$, ${\text{Zr-d}}_{{\text{z}}^{2}}$ orbitals for the pure Zr system were exhibited. In region A1, the point P is mainly coming from Zr-d_xz_ and Zr-d_xy_ orbitals, however, the point Q is mainly arising from the Zr-d_yz_ and ${\text{Zr-d}}_{{\text{x}}^{2}-{\text{y}}^{2}}$ orbitals. In region A2, the surface states (*k*_*z*_ = π plane) are dominated by the ${\text{Zr-d}}_{{\text{z}}^{2}}$ orbital, however, the contribution of other orbitals of the Zr atom cannot be ignored.

## Data Availability Statement

All datasets generated for this study are included in the article/Supplementary Material.

## Author Contributions

LZ: conceptualization, methodology, software, formal analysis, data curation, and writing. KW: investigation, funding, and project administration. All authors contributed to the article and approved the submitted version.

## Conflict of Interest

The authors declare that the research was conducted in the absence of any commercial or financial relationships that could be construed as a potential conflict of interest.

## Footnotes

^1^Available online at: https://materialsproject.org/materials/mp-131/

## References

[B1] Alisultanov Z. Z. (2018). Hybrid Weyl semimetal under crossed electric and magnetic fields: field tuning of spectrum type. Phys. Lett. A 382, 3211–3215. 10.1016/j.physleta.2018.08.028

[B2] Chen H. Zhang S. Jiang W. Zhang C. Guo H. Liu Z. (2018). Prediction of two-dimensional nodal-line semimetals in a carbon nitride covalent network. J. Mater. Chem. 6, 11252–11259. 10.1039/C8TA02555J

[B3] Fang C. Lu L. Liu J. Fu L. (2016). Topological semimetals with helicoid surface states. Nat. Phys. 12, 936–941. 10.1038/nphys3782

[B4] Fu C. Guin S. N. Watzman S. J. Li G. Liu E. Kumar N. (2018). Large Nernst power factor over a broad temperature range in polycrystalline Weyl semimetal NbP. Energy Environ. Sci. 11, 2813–2820. 10.1039/C8EE02077A

[B5] Gan L. Wang R. Jin Y. J. Ling D. B. Zhao J. Xu W. P.. (2017). Emergence of topological nodal loops in alkaline-earth hexaborides XB_6_ (X = Ca, Sr, and Ba) under pressure. Phys. Chem. Chem. Phys. 19, 8210–8215. 10.1039/C6CP08421D 28271108

[B6] Gao H. Venderbos J. W. Kim Y. Rappe A. M. (2019). Topological semimetals from first-principles. Ann. Rev. Mater. Res. 49, 153–183. 10.1146/annurev-matsci-070218-010049

[B7] Gao Y. Xie Y. Chen Y. Gu J. Chen Z. (2018). Spindle nodal chain in three-dimensional α′ boron. Phys. Chem. Chem. Phys. 20, 23500–23506. 10.1039/C8CP03874K 30183022

[B8] Hu J. Xu S. Ni N. Mao Z. (2019). Electronic transport and quantum oscillation of topological semimetals. Ann. Rev. Mater. Res. 49, 207–252. 10.1146/annurev-matsci-070218-010023

[B9] Jin L. Zhang X. He T. Meng W. Dai X. Liu G. (2019). Topological nodal line state in superconducting NaAlSi compound. J. Mater. Chem. C 7, 10694–10699. 10.1039/C9TC03464A

[B10] Jin L. Zhang X. He T. Meng W. Dai X. Liu G. (2020). Electronic structure, doping effect and topological signature in realistic intermetallics Li_3−x_Na_x_M (x = 3, 2, 1, 0; M = N, P, As, Sb, Bi). Phys. Chem. Chem. Phys. 22, 5847–5854. 10.1039/C9CP06033B 32107508

[B11] Jin Y. J. Wang R. Zhao J. Du Y. Zheng C. Gan L.. (2017). The prediction of a family group of two-dimensional node-line semimetals. Nanoscale 9, 13112–13118. 10.1039/C7NR03520A 28849838

[B12] Klemenz S. Lei S. Schoop L. M. (2019). Topological semimetals in square-net materials. Ann. Rev. Mater. Res. 49, 185–206. 10.1146/annurev-matsci-070218-01011432142261

[B13] Koepernik K. Kasinathan D. Efremov D. V. Khim S. Borisenko S. V. Buchner B. (2016). TaIrTe_4_: a ternary type-II Weyl semimetal. Phys. Rev. B 93:201101 10.1103/PhysRevB.93.201101

[B14] Liang Q. Zhou J. Yu R. Wang Z. Weng H. (2016). Node-surface and node-line fermions from nonsymmorphic lattice symmetries. Phys. Rev. B 93:085427 10.1103/PhysRevB.93.085427

[B15] Liu J. Li X. Wang Q. Kawazoe Y. Jena P. (2018). A new 3D Dirac nodal-line semi-metallic graphene monolith for lithium ion battery anode materials. J. Mater. Chem. 6, 13816–13824. 10.1039/C8TA04428G

[B16] Liu P. Zhou L. Tretiak S. Wu L. (2017). Two-dimensional hexagonal M3C2 (M = Zn, Cd and Hg) monolayers: novel quantum spin Hall insulators and Dirac cone materials. J. Mater. Chem. C 5, 9181–9187. 10.1039/C7TC02739G

[B17] Lu J. Luo W. Li X. Yang S. Cao J. Gong X. (2017). Two-dimensional node-line semimetals in a honeycomb-kagome lattice. Chin. Phys. Lett. 34:057302 10.1088/0256-307X/34/5/057302

[B18] Ma J. Gu Q. Liu Y. Lai J. Yu P. Zhuo X.. (2019). Nonlinear photoresponse of type-II Weyl semimetals. Nat. Mater. 18, 476–481. 10.1038/s41563-019-0296-5 30833780

[B19] Meng L. Li Y. Wu J. Zhao L. Zhong J. (2020a). A type of novel Weyl semimetal candidate: layered transition metal monochalcogenides Mo_2_XY (X, Y = S, Se, Te, X ≠ Y). Nanoscale 12, 4602–4611. 10.1039/C9NR09123H 32043508

[B20] Meng L. Wu J. Zhong J. Romer R. A. (2019). A type of robust superlattice type-I Weyl semimetal with four Weyl nodes. Nanoscale 11, 18358–18366. 10.1039/C9NR04551A 31573592

[B21] Meng W. Zhang X. He T. Jin L. Liu G. (2020b). Multiple fermionic states with clear nontrivial surface signature in CsCl-type compound eras. Comput. Mater. Sci. 183:109815 10.1016/j.commatsci.2020.109815

[B22] Osterhoudt G. Diebel L. K. Gray M. Yang X. Stanco J. Huang X.. (2019). Colossal mid-infrared bulk photovoltaic effect in a type-I Weyl semimetal. Nat. Mater. 18, 471–475. 10.1038/s41563-019-0297-4 30833781

[B23] Ouyang T. Xiao H. Tang C. Hu M. Zhong J. (2016). Anisotropic thermal transport in Weyl semimetal TaAs: a first principles calculation. Phys. Chem. Chem. Phys. 18, 16709–16714. 10.1039/C6CP02935C 27271203

[B24] Perdew J. P. Burke K. Ernzerhof M. (1996). Generalized gradient approximation made simple. Phys. Rev. Lett. 77:3865. 10.1103/PhysRevLett.77.3865 10062328

[B25] Perdew J. P. Burke K. Ernzerhof M. (1998). Perdew, Burke, and Ernzerhof reply. Phys. Rev. Lett. 80:891 10.1103/PhysRevLett.80.891

[B26] Pham A. Klose F. Li S. (2019). Robust topological nodal lines in halide carbides. Phys. Chem. Chem. Phys. 21, 20262–20268. 10.1039/C9CP04330F 31490493

[B27] Phillips M. R. Aji V. (2014). Tunable line node semimetals. Phys. Rev. B 90:115111 10.1103/PhysRevB.90.115111

[B28] Schoop L. M. Pielnhofer F. Lotsch B. V. (2018). Chemical principles of topological semimetals. Chem. Mater. 30, 3155–3176. 10.1021/acs.chemmater.7b05133

[B29] Sharma G. Goswami P. Tewari S. (2017). Chiral anomaly and longitudinal magnetotransport in type-II Weyl semimetals. Phys. Rev. B 96:045112 10.1103/PhysRevB.96.045112

[B30] Soluyanov A. A. Gresch D. Wang Z. Wu Q. Troyer M. Dai X.. (2015). Type-II Weyl semimetals. Nature 527, 495–498. 10.1038/nature15768 26607545

[B31] Sun G. Kurti J. Rajczy P. Kertesz M. Hafner J. Kresse G. (2003). Performance of the Vienna ab initio simulation package (VASP) in chemical applications. J. Mol. Struct-Theochem. 624, 37–45. 10.1016/S0166-1280(02)00733-9

[B32] Wang X. Cheng Z. Zhang G. Wang B. Wang X. Chen H. (2020). Rich novel zero-dimensional (0D), 1D, and 2D topological elements predicted in the P63/m type ternary boride HfIr_3_B_4_. Nanoscale 12:8314. 10.1039/D0NR00635A 32236236

[B33] Wu W. Liu Y. Li S. Zhong C. Yu Z. Sheng X. (2018). Nodal surface semimetals: theory and material realization. Phys. Rev. B 97:115125 10.1103/PhysRevB.97.115125

[B34] Wyckoff R. W. G. (1963). New York Hexagonal Closest Packed, Hcp, Structure Crystal Structures. 2nd ed (New York, NY: Interscience Publishers), 7–83.

[B35] Xie H. Qie Y. Imran M. Sun Q. (2019). Topological semimetal porous carbon as a high-performance anode for Li-ion batteries. J. Mater. Chem. A, 7, 14253–14259. 10.1039/C9TA03587G

[B36] Yan B. Felser C. (2017). Topological materials: weyl semimetals. Ann. Rev. Condensed Matter Phys. 8, 337–354. 10.1146/annurev-conmatphys-031016-025458

[B37] Yang B. Zhang X. Zhao M. (2017). Dirac node lines in two-dimensional Lieb lattices. Nanoscale 9, 8740–8746. 10.1039/C7NR00411G 28616940

[B38] Yi X. Li W. Q. Li Z. H. Zhou P. Ma Z. Sun L. Z. (2019). Topological dual double node-line semimetals NaAlSi(Ge) and their potential as cathode material for sodium ion batteries. J. Mater. Chem. C 7, 15375–15381. 10.1039/C9TC04096J

[B39] Yu Z. Yao Y. Yang S. A. (2016). Predicted unusual magnetoresponse in type-II weyl semimetals. Phys. Rev. Lett. 117:077202. 10.1103/PhysRevLett.117.077202 27563994

[B40] Zhang M. Zhang S. Wang P. Zhang C. (2020). Emergence of a spin-valley Dirac semimetal in a strained group-VA monolayer. Nanoscale 12, 3950–3957. 10.1039/C9NR09545D 32010916

[B41] Zhang X. Yu Z. Sheng X. Yang H. Y. Yang S. A. (2017). Coexistence of four-band nodal rings and triply degenerate nodal points in centrosymmetric metal diborides. Phys. Rev. B 95:235116. 10.1103/PhysRevB.95.23511629129074

[B42] Zhang X. Yu Z. Zhu Z. Wu W. Wang S. Sheng X. (2018). Nodal loop and nodal surface states in the Ti3Al family of materials. Phys. Rev. B 97:235150 10.1103/PhysRevB.97.235150

[B43] Zhong C. Chen Y. Xie Y. Yang S. A. Cohen M. L. Zhang S. B. (2016). Towards three-dimensional Weyl-surface semimetals in graphene networks. Nanoscale 8, 7232–7239. 10.1039/C6NR00882H 26971563

[B44] Zhou P. Ma Z. Sun L. Z. (2018). Coexistence of open and closed type nodal line topological semimetals in two dimensional B2C. J. Mater. Chem. C, 6, 1206–1214. 10.1039/C7TC05095J

[B45] Zhou T. Zhang C. Zhang H. Xiu F. Yang Z. (2016). Enhanced thermoelectric properties of the Dirac semimetal Cd_3_As_2_. Inorg. Chem. Front. 3, 1637–1643. 10.1039/C6QI00383D

