# Supplementary material for: Pure Zirconium: Type II Nodal Line and Nodal Surface States
Source: Front Chem. 2020 Sep 23;8:585753. doi: 10.3389/fchem.2020.585753 (PMC7538698; doi:10.3389/fchem.2020.585753)
Supplement: Supplementary file 1 [file Table_1.doc]

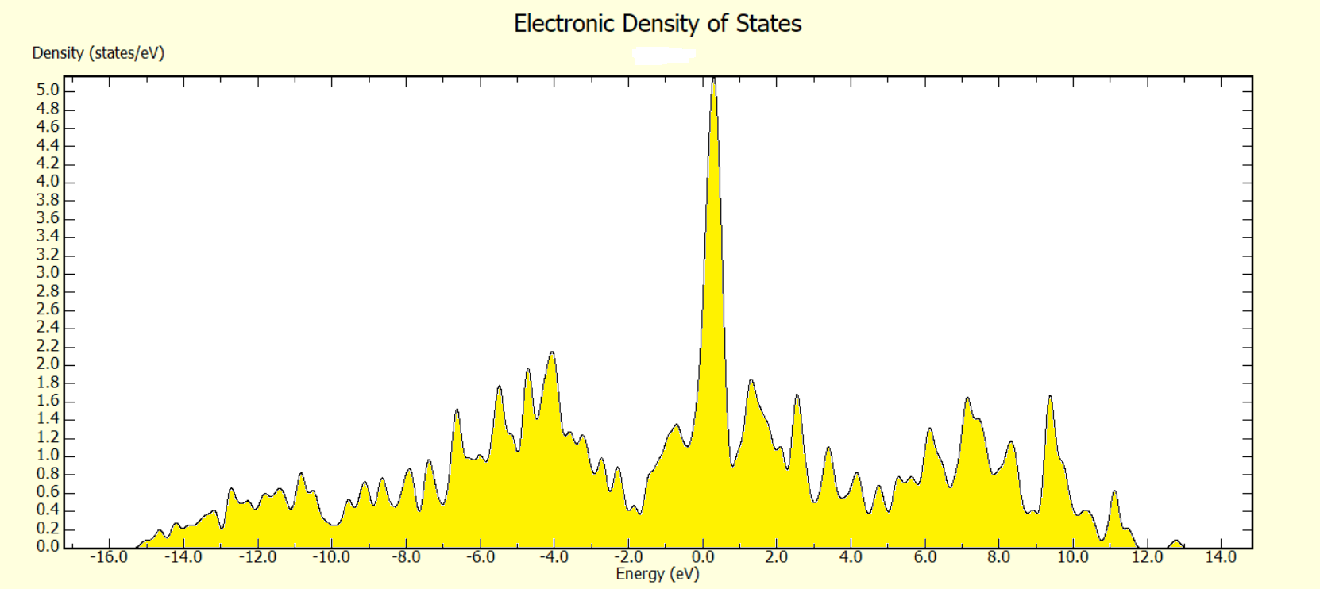


Figure S1 Electronic density of states of Zr metal.


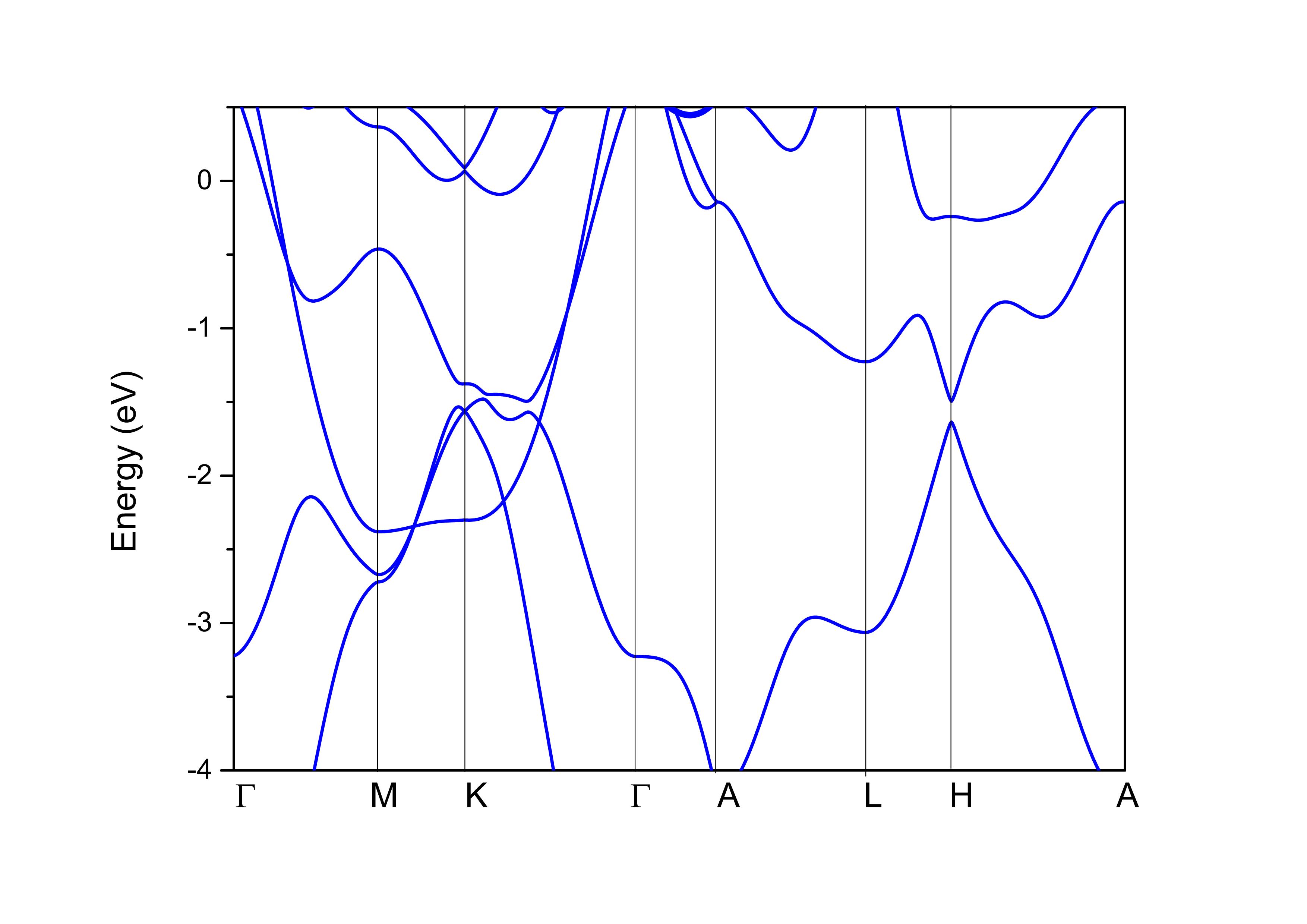


Figure S2 Electronic band structure of Zr metal at its experimental lattice constants.
